# Cultivating Andrija Štampar’s legacy: from international health and positive health to person-centered care

**DOI:** 10.3325/cmj.2023.64.444

**Published:** 2023-12

**Authors:** Juan E. Mezzich, Marijana Braš, Veljko Đorđević, Slavko Orešković, Neda Pjevač, Snježana Kaštelan

**Affiliations:** 1 Icahn School of Medicine at Mount Sinai, New York, NY, USA; 2 Hipolito Unanue Chair of Person-Centered Medicine, San Marcos National University, Lima, Peru; 3 International College of Person-Centered Medicine; 4 Department of Medical Ethics and Communication Skills, Center for Palliative Medicine, University of Zagreb School of Medicine, Zagreb, Croatia; 5 Department of Obstetrics and Gynecology, University Hospital Center Zagreb, University of Zagreb School of Medicine, Zagreb, Croatia; 6 Department of Medical Statistics, Epidemiology and Medical Informatics, University of Zagreb School of Medicine, Andrija Štampar School of Public Health, Zagreb, Croatia; 7 Department of Ophthalmology, University Hospital Dubrava, University of Zagreb School of Medicine, Zagreb, Croatia

Andrija Štampar was born in 1888 in the village of Drenovac in Croatia, but owing to his vocation and the reach of his ideas and commitments, he belongs to the world. Štampar is known for pioneering international public health work and successful collaborative projects in Yugoslavia and China. Due to his multi-year efforts to conceptualize and build the World Health Organization (WHO), he is often regarded as its father. The perspectives and personal examples that Štampar offered opened new paths for the understanding of health and the practice of health actions, projecting a legacy that remains vivid and inspiring to this day (1).

## Andrija Štampar's role in the establishment of the World Health Organization

The establishment of an international health organization required a pioneer with the ability to seek, engage, and persuade leaders and peoples across all continents. This person needed to possess extraordinary intellect, interpersonal skills, conviction, and persistence. Andrija Štampar, perhaps better than anybody else, fit this description. He labored and traveled for many years to bring this dream to reality (1). Unsurprisingly, Štampar was appointed the president of the first World Health Assembly, and as such he had a crucial role in the drafting of the WHO Constitution (2).

## Andrija Štampar and the definition of health

The concepts of health in ancient civilizations, from the Chinese and Ayurvedic to Hellenic, African, and Andean American always involved personalization and a holistic framework (3). It is with this background that Andrija Štampar at the first World Health Assembly crafted a definition of health. It reads, “*Health is the state of complete physical, emotional, and social well-being, and not merely the absence of illness or infirmity.”* This definition is enshrined in the Constitution of the WHO (2) and represents one of the best-known contributions from Štampar to the world.

## Andrija Štampar and the focus on positive health

The WHO's health definition is formulated in terms of positive health, rather than focusing on ill-health, which conventionally garners more attention in its various forms (diseases, disabilities, distress). While the conceptual framework of positive health encompasses a variety of elements, including adaptive functioning, resilience, as well as emotional and instrumental resources, its most comprehensive and significant component is the quality of life or well-being (4,5). In fact, the WHO's definition of health accentuates well-being.

## Andrija Štampar and people-centered care

Another broad and important health perspective pioneered by Štampar was that of people-centered care. The elements of his concern include the following: a) Focusing on educating people is of greater significance than enforcing laws. b) Public health and its enhancement should not be controlled solely by medical authorities. Instead, there should be a collective effort, since improvements in health can only be achieved through collaboration. c) A physician should primarily function as a social worker since individual therapy alone may not yield significant results. In fact, social therapy should be the key to success. d) It is essential to establish a health care organization where physicians actively reach out to patients, rather than patients to physicians. This approach will ensure the inclusion of a growing number of individuals who require health care services. e) The physician must assume the role of an educator for the community. f) The primary area where physicians should focus their efforts is the community, rather than exclusively laboratories and private examination rooms (6).

## Štampar’s legacy and the flourishment of positive health

Since Štampar’s time, interest in positive health has been growing steadily. One major reflection of this is the development of health promotion. In line with the WHO's Charter for Health Promotion (1986), health is established and experienced by individuals in their daily lives, encompassing their learning, employment, leisure activities, and relationships. It is fostered through self-care and care for others. Individuals should have the capacity to make choices and exert control over one's life, and society should create an environment that enables all its members to achieve good health (7).

The use of positive health and well-being concepts is increasing in a wide variety of health fields (4,8). Implementation of projects and services has been facilitated by the design and validation of solid generic instruments. Among the most widely used are the WHO Quality of Life Instrument (9) and the Multicultural Quality of Life Index (10).

The importance ascribed to positive health is growing internationally. This is illustrated by the United Nations’ (2015) Sustainable Development Goals Declaration, which formulated its Goal 3 on Health in terms of ensuring well-being and promoting health for individuals of all age groups (11). Even large countries that reacted modestly to early declarations, such as the Alma-Ata Declaration, are now responding enthusiastically to the Sustainable Development Goals.

## Štampar’s legacy and the emergence of person-centered medicine and health

Andrija Štampar's views on positive health and patient-centered care came to the forefront in the 20th century, a period marked by significant advancements in scientific medicine, which emphasized organs and diseases. While this approach brought about notable progress in diagnosis and treatment, it also led to the deterioration of the doctor-patient relationship, the dehumanization of medical practice, and the commercialization of health care (12).

In reaction to the prevalent reductionist medicine, early person-centered formulations appeared in various countries during the 20th century. The Spanish philosopher Jose Ortega y Gasset (1914) offered his maxim: “*I am I and my circumstance, and if I do not save it, I do not save myself”* (13). Paul Tournier, a family doctor in Geneva published *Médecine de la Personne* (1940) (14). In the United States, the educator and psychologist Carl Rogers published *Becoming a Person* (1961) (15) and *The Person as Center* (1981) (16). Additionally, there were proposals for patient- or person-centered medicine linked to key medical areas such as dementia (17) and family medicine (18). Further, advocacy efforts in specific countries, such as Italy, proposed changes related to medical epistemology (19).

The 21st century is increasingly acknowledged as the Century of the Person, with a special emphasis on the fields of medicine and health. The concept of “person-centered medicine” was established as a global initiative by the International College of Person-Centered Medicine in partnership with the World Medical Association, WHO, International Council of Nurses, and 30 other organizations during a decade and a half of annual Geneva Conferences. It emphasizes the individual as the core of health care, striving to make the person the primary focus and driving force behind health-related initiatives. Prioritizing the individual in medicine entails a patient-centered approach, where medicine is oriented toward the individual, with the individual, for the individual, and in collaboration with the individual. By integrating science and humanism, this approach aspires to create a medical practice that is guided by evidence, personal experiences and values, ultimately dedicated to the enhancement of health and well-being for all (20).

Major publications from the International College of Person-Centered Medicine include *Person Centered Psychiatry* (21), *Seeking the Person at the Center of Medicine* (22), and *Person Centered Medicine* (20). Another book cultivating Štampar’s legacy is *Person in Medicine and Health Care: From Bench to Bedside to Community* by Đorđević, Braš, and Miličić published in Croatia in 2012 (6).

## Horizons toward “whole person,” “whole health,” and “whole care”

Effective communication is vital for promoting healthy aging at all levels, locally and globally (23). The twentieth-century medicine specialized deeply within each field, but the twenty-first-century medicine aims for integration, achievable through person-centered education. The COVID-19 pandemic has highlighted the importance of adaptive teaching methods, including distance learning. Our world is at a historic turning point, and an opportunity is being offered to create innovative education models for future health care professionals facing diverse challenges. Building a global network of educators dedicated to creating a person-centered medical curriculum is of utmost importance (24). Personalized medicine focuses on science, while person-centered medicine emphasizes holistic care. Despite their differences, they are not mutually exclusive but share a strong bond. Person-centered medicine addresses the shortcomings of fragmented, organ-specific care. Healthcare professionals should use effective communication, build relationships with patients, and recognize the uniqueness of each patient. This approach complements evidence-based medicine, ensuring a balanced patient-centered practice (25).

Extending Štampar’s legacy, we are witnessing the emergence of new developments in which the concept of “whole person” as a multidimensional, indivisible, and irreducible being is being articulated, together with those of “total health” and “total care” (26).

“Total health” is emerging in reference to all that needs to be considered for a thorough understanding of health and the planning of effective health actions. One factor here is the importance of considering both ill-health (diseases, distress, disabilities) and positive health (resources, resilience, well-being). Another is the growing acceptance of the multidimensionality of health, from the conventional bio-psycho-social to the more encompassing bio-psycho-spiritual and eco-social framework.

The argumentation for “total care” includes, first, comorbidity and multimorbidity as prevalent and ever-growing realities in the clinical arena. Syndemics research is demonstrating the importance of attending to the interrelations between coexisting clinical conditions and the interrelations between them and their contextual factors. Since the Declarations of Alma Ata (27) and Astana (28), it has been recognized that the domain of care is broader than just professional services as it should include integral care by all for all. Care focused exclusively on specific disorders is insufficiently effective, and attention should also be paid to cross-sectional context and longitudinal dynamic development.

Understanding health and organizing care as a holistic concept seems to maximize their relevance and effectiveness in achieving the well-being of individuals and communities. This is increasingly recognized as the fundamental purpose of medicine, health care, education, and social governance.
